# Dietary glutamine modulates egg albumen and yolk composition in laying quails: effects of age

**DOI:** 10.1016/j.psj.2026.107418

**Published:** 2026-07-12

**Authors:** Ewa Tomaszewska, Kornel Kasperek, Kamil Drabik, Iwona Puzio, Katarzyna J. Zelewska, Justyna Batkowska

**Affiliations:** a Department of Animal Physiology, University of Life Sciences in Lublin, Akademicka 12, 20-950 Lublin, Poland; b Institute of Biological Basis of Animal Production, University of Life Sciences in Lublin, Akademicka 13, 20-950, Lublin, Poland; c Institute of Basic Medical Sciences, Department of Comparative Medicine, University of Oslo, Sognsvannsveien 9, 0372 Oslo, Norway

**Keywords:** Quail, Egg, Amino acid profile, Age, Amino acids supplementation

## Abstract

This study evaluated the effects of dietary l-glutamine (Gln) supplementation on egg composition and amino acid profiles in laying Japanese quail (Coturnix japonica). A total of 320 birds were assigned to four dietary treatments containing 0.0%, 0.5%, 1.0%, or 1.5% Gln for 12 wk. Eggs were collected at 12 and 19 wk of age, representing the early and late stages of the peak laying period, for analyses of yolk and albumen composition. Dietary Gln supplementation significantly affected the concentration of several amino acids in both yolk and albumen, although the response depended on bird age and Gln inclusion level. The effects were more pronounced in late peak-lay birds and were generally greatest at moderate supplementation levels (0.5% or 1.0% Gln). In albumen, increasing dietary Gln was associated with selective changes in amino acid composition, whereas yolk amino acid responses showed more evident age-dependent patterns. Proximate composition of egg fractions was affected to a lesser extent than amino acid profiles. Overall, the results indicate that dietary Gln modulates amino acid deposition in egg components in an age-dependent manner rather than uniformly enhancing egg nutritional value. This study demonstrates for the first time that dietary Gln supplementation can alter yolk and albumen amino acid profiles in laying quail, supporting its role as a dietary factor influencing amino acid deposition during different stages of lay.

## Introduction

Egg quality and composition are of considerable importance in poultry nutrition and human diets. Eggs are recognized as a valuable source of high-quality protein and essential nutrients and represent one of the most affordable animal-derived foods worldwide (Walker and Baum, 2022). Beyond production performance, increasing attention has been directed toward internal egg quality, particularly the chemical composition of albumen and yolk, because these traits determine both nutritional and technological properties of eggs (Nys et al., 2011). Consequently, dietary strategies capable of modulating egg composition, including amino acid profiles, are of growing interest in poultry nutrition.

L-Glutamine (Gln) is traditionally classified as a non-essential amino acid; however, under conditions of elevated metabolic demand, including rapid growth, stress, or intensive egg production, it may become conditionally essential (Wu, 2013). Glutamine plays important roles in nitrogen metabolism and serves as a major energy substrate for enterocytes and immune cells, thereby contributing to intestinal function and physiological homeostasis. In poultry, dietary Gln supplementation has been associated with alterations in growth performance, intestinal morphology, nutrient utilization, and selected production traits (Bartell and Batal, 2007). Previous studies also suggest that dietary inclusion levels around 1% may influence physiological and productive responses in poultry, although these effects appear to depend on species, physiological status, and experimental conditions (Tomaszewska et al., 2025).

Recent studies in Japanese quails demonstrated that dietary Gln supplementation may influence several physiological and productive parameters. Tomaszewska et al. (2025) reported that dietary inclusion of 0.5-1.5% Gln affected laying performance, intestinal morphology, blood biochemical indices, and gut microbiota composition in laying quails, with some responses differing between early and later stages of lay. Previous studies from the same experimental model demonstrated effects of dietary glutamine on fatty acid composition and lipid-related health indices in quail meat, and bone quality traits in laying quails. Collectively, these studies indicate that Gln may act as a functional metabolic modulator in quail physiology.

Although the effects of dietary glutamine supplementation on selected egg quality traits have been investigated in laying hens, comparable data for Japanese quail remain unavailable. Given the substantial species-specific differences in metabolic rate, reproductive physiology, nutrient partitioning, and production characteristics between hens and quail, findings obtained in laying hens cannot be directly extrapolated to quail. Consequently, the effects of dietary glutamine on egg composition and amino acid deposition in laying Japanese quail remain largely unexplored, despite increasing interest in functional amino acid supplementation in poultry nutrition. Moreover, because egg composition changes during the laying cycle, bird age may influence the metabolic response to dietary Gln supplementation and nutrient partitioning into egg components.

Therefore, the present study aimed to evaluate the effects of dietary Gln supplementation on proximate composition and amino acid profiles of egg albumen and yolk in laying Japanese quails at different stages of lay. We hypothesized that dietary Gln supplementation would modulate egg composition and amino acid deposition in an age-dependent manner.

## Materials and methods

### *Ethical approval*

Approval from the Local Ethics Committee for Animal Experiments in Lublin, Poland (resolution No. 40/2023) was obtained before the research commenced.

### *Birds, experimental diets and eggs collection*

The experimental procedures, housing conditions, and husbandry practices were described previously (Tomaszewska et al., 2025). The study was designed to evaluate the effects of dietary Gln supplementation on egg composition in laying Japanese quails during the established laying phase.

At 6 wk of age, a total of 320 healthy female Japanese quails (Coturnix japonica) were allocated to 4 dietary treatments in a completely randomized design with 8 replicate cages per treatment and 10 birds per cage. Birds were housed in individual layer cages equipped with trough feeders and nipple drinkers and maintained under standard environmental and lighting conditions appropriate for laying quails.

A basal wheat-soybean meal diet formulated to meet the nutrient requirements of laying quails served as the control diet. Experimental diets were prepared by supplementing the basal diet with 0%, 0.5%, 1.0%, or 1.5% free l-glutamine (Biomus, Lublin, Poland; purity ≥99.5%). All diets were provided in mash form, and birds had ad libitum access to feed and water throughout the experiment. The basal diet was analyzed for its total glutamine/glutamate equivalents originating from dietary protein using ion-exchange chromatography (AAA 400 amino acid analyzer, INGOS, Prague, Czech Republic), following AOAC method 994.12. The value obtained was 32 g/kg feed and represents total glutamine/glutamate equivalents after protein hydrolysis, not free crystalline l-glutamine. Free l-glutamine was supplied only through dietary supplementation at 0.5%, 1.0%, or 1.5%. The composition and nutrient content of all experimental diets are presented in Table 1. The feeding period lasted 12 wk, from 7 to 19 wk of age.

**Table 1:** Composition and nutrient content of experimental diets, % dry matter.

	diets			
Ingredients, %	0% Gln	0.5% Gln	1% Gln	1.5% Gln
Soybean meal	30.40	30.40	30.40	30.40
Wheat	26.65	26.65	26.65	26.65
Corn	14	14	14	14
Triticale	10	10	10	10
Limestone	5.18	5.18	5.18	5.18
Wheat bran	5	5	5	5
Soybean oil	4.30	4.30	4.30	4.30
Monocalcium phosphate	1.95	1.95	1.95	1.95
NaCl	0.36	0.36	0.36	0.36
DL-Methionine	0.08	0.08	0.08	0.08
L-lysine	0.08	0.08	0.08	0.08
Vitamin-mineral premix^1^	0.5	0.5	0.5	0.5
L-glutamine	0	0.5	1.0	1.5
Kaolin	1.5	1.0	0.5	0.0
*Nutrients composition, calculated*				
Metabolizable energy, kcal/kg	2800			
Crude protein	21			
Crude fiber	2.954			
Arginine	1.396			
Lysine	1.15			
Methionine	0.384			
Methionine +cysteine	0.75			
Treonine	0.78			
Ca	2.5			
Total P	0.858			
Available P	0.55			
Na	0.16			

1 The premix provided per 1 kg of diet: vitamin A, 10,000 IU (retinol); vitamin D3, 2,500 IU (cholecalciferol); vitamin E, 30 IU (α-tocopherol); 6-phytase, 1500 FTU; Endo-1,4-beta-xylanase 2000 IU.

Eggs were collected after 6 and 12 wk of dietary supplementation, corresponding to 12 and 19 wk of age, respectively. At each sampling point, eggs were collected from each replicate cage over 2 consecutive days. Because quails were housed in groups and individual oviposition was not monitored, the eggs could not be assigned to specific females. Therefore, egg sampling was performed at the replicate-cage level. From the total pool of intact eggs collected from each cage during the 2-day sampling period, 10 eggs were randomly selected for subsequent analyses. For proximate composition and amino acid analyses, albumens and yolks from these 10 eggs were separated and pooled separately to obtain 1 composite albumen sample and 1 composite yolk sample per replicate cage. The replicate cage was considered the experimental unit.

### Sample storage and proximate composition analysis

All pooled samples were homogenized and stored at −20°C until analysis. Before chemical analyses, samples were thawed at 4°C and thoroughly mixed to ensure sample uniformity.

Chemical composition of egg albumen and yolk was determined according to standard methods of the Association of Official Analytical Chemists (AOAC, 2019). Moisture content was determined by oven drying at 103 ± 2°C to constant weight (AOAC method 934.01). Crude protein content was determined using the Kjeldahl method (AOAC method 954.01), with nitrogen converted to protein using a conversion factor of 6.25. Crude fat content was determined by ether extraction (AOAC method 920.39), whereas ash content was measured after incineration in a muffle furnace at 550°C (AOAC method 942.05).

Available carbohydrates were calculated by difference. Energy value of egg albumen and yolk (kJ/100 g) was calculated using Atwater conversion factors based on the determined chemical composition.

### Amino acid analysis

The amino acid composition of egg white and yolk was determined according to AOAC methods for total amino acid quantification (AOAC, 2019). Samples were subjected to acid hydrolysis with 6 N HCl at 110°C for 20 h under oxygen-free conditions (AOAC method 994.12). Sulfur-containing amino acids (methionine, cysteine) were analyzed after performic acid oxidation prior to hydrolysis, and tryptophan was quantified following alkaline hydrolysis with barium hydroxide (AOAC method 988.15). Amino acids were separated and quantified using ion-exchange chromatography with post-column derivatization and ninhydrin detection. Concentrations were calculated using certified external amino acid standards.

### *Statistical analysis*

Statistical analyses were performed using the UNIVARIATE and GLM procedures of SAS software (Statistical Analysis System, version 9.4; SAS Institute Inc., Cary, NC, USA). The normality of data distribution was verified using the Kolmogorov-Smirnov test.

Data were analyzed using a two-way analysis of variance with age of birds (2 levels: 12 and 19 wk) and dietary Gln dose (4 levels: 0, 0.5, 1.0, and 1.5%) as fixed effects, including their interaction. The replicate cage was considered the experimental unit.

Orthogonal polynomial contrasts were applied to evaluate linear and quadratic responses to increasing dietary Gln levels. When significant main effects or interactions were detected, differences among least-squares means were assessed using Tukey’s multiple comparison test. Statistical significance was declared at *P* ≤ 0.05.

Table 1, Table 2, Table 3 present least-squares means for the main effects of age and dietary Gln dose, along with P-values for the main effects, their interaction, and the orthogonal linear and quadratic contrasts within the dose effect. Table 4, Table 5, Table 6, Table 7 present least-squares means for traits exhibiting a significant age × dose interaction (*P* < 0.05), with detailed values for each treatment combination.

**Table 2:** Proximate composition of egg albumen as affected by main factors.

TRAIT^1^	Age			Dose					Effect of			Contrast^2^	
	12	19	SEM	0	0.5	1	1.5	SEM	age	dose	age x dose	linear	quadratic
Moisture (%)	86.7^b^	88.3^a^	0.32	87.2	86.9	87.9	87.8	0.46	0.001	0.38	0.49	0.22	0.92
Crude protein (%)	10.9^a^	9.4^b^	0.22	10.9^a^	10.4^ab^	9.8^ab^	9.6^b^	0.32	<0.001	0.02	0.13	0.01	0.72
Crude fat (%)	0.10	0.077	0.02	0.05	0.12	0.08	0.12	0.02	0.23	0.13	0.17	0.17	0.59
Ash (%)	0.94^a^	0.74^b^	0.03	0.88	0.89	0.84	0.76	0.04	<0.001	0.08	0.38	0.05	0.32
Available carbohydrates (%)	1.34	1.39	0.14	1.04	1.49	1.22	1.71	0.20	0.78	0.11	0.005	0.08	0.93
Energy (kJ/100 g)	213^a^	187^b^	3.49	205	207	192	196	4.9	<0.001	0.12	0.05	0.13	0.85

1 Data are presented as least squares means and standard error of the mean (SEM) ^a,b^ Means in the same row and for the same main effect (age or dose) with different superscripts are significantly different (*P* < 0.05, Tukey’s HSD test).

2 Linear or quadratic effects were evaluated by orthogonal polynomial contrasts to test the effects of Gln levels on selected traits.

**Table 3:** Proximate composition of egg yolk as affected by main factors.

TRAIT^1^	Age			Dose					Effect of			Contrast^2^	
	12	19	SEM	0	0.5	1	1.5	SEM	age	dose	age x dose	linear	quadratic
Moisture (%)	52.7^a^	42.9^b^	1.20	50.0^a^	49.2^a^	50.2^a^	41.9^b^	1.7	<0.001	0.002	0.01	0.02	0.09
Crude protein (%)	15.9^b^	16.8^a^	0.18	15.9^b^	17.7^a^	16.2^b^	15.5^b^	0.25	0.001	<0.001	0.57	0.02	<0.001
Crude fat (%)	24.8^b^	28.9^a^	0.61	27.6^ab^	26.7^ab^	24.5^b^	28.5^a^	0.87	<0.001	0.01	0.03	0.91	0.02
Ash (%)	1.9^b^	2.2^a^	0.03	2.1^a^	2.2^a^	1.9^b^	2.1^ab^	0.05	<0.001	0.001	<0.001	0.18	0.76
Available carbohydrates (%)	3.5^b^	5.3^a^	0.27	4.3	4.3	4.8	4.1	0.38	<0.001	0.54	0.03	0.94	0.39
Energy (kJ/100 g)	1289^b^	1441^a^	10.2	1366	1358	1353	1383	14.41	<0.001	0.48	0.001	0.69	0.45

1 Data are presented as least squares means and standard error of the mean (SEM) ^a, b^, Means in the same row and for the same main effect (age or dose) with different superscripts are significantly different (*P* < 0.05, Tukey’s HSD test).

2 Linear or quadratic effects were evaluated by orthogonal polynomial contrasts to test the effects of Gln levels on selected traits.

**Table 4:** Proximate composition of egg albumen and yolk for traits showing a significant age × dose interaction.

Age	Dose	TRAIT^1^					
		Moisture (%)-Yolk	Crude fat (%)-Yolk	Ash (%)-Yolk	Available carbohydrates (%)- Albumen	Available carbohydrates (%)- Yolk	Energy (kJ/100 g)- Yolk
12	0	54.1^b^	25.7^ab^	2.1^bc^	1.6^ab^	2.5^c^	1257^d^
	0.5	52.6^b^	24.7^ab^	2.0^c^	1.4^ab^	3.6^abc^	1261^d^
	1	52.5^b^	20.5^b^	1.8^cd^	1.1^ab^	4.7^abc^	1285^cd^
	1.5	51.7^b^	28.1^a^	1.7^d^	1.2^ab^	3.0^bc^	1354^bc^
19	0	45.9^b^	29.6^a^	2.1^bc^	0.5^b^	5.9^a^	1476^a^
	0.5	45.7^b^	28.6^a^	2.4^ab^	1.6^ab^	5.0^ab^	1454^a^
	1	47.8^b^	28.5^a^	2.0^c^	1.3^ab^	5.0^ab^	1422^ab^
	1.5	32.1^a^	28.9^a^	2.4^a^	2.2^a^	5.2^ab^	1412^ab^
SEM		2.4	1.2	0.068	0.283	0.534	20.4

1 Data are presented as least squares means and standard error of the mean (SEM) ^a, b, c, d^ Means in the same row and for the same main effect (age or dose) with different superscripts are significantly different (*P* < 0.05, Tukey’s HSD test).

**Table 5:** Amino acid composition of egg albumen (mg/g) as affected by main factors.

TRAIT^1^	Age			Dose					Effect of			Contrast^2^	
	12	19	SEM	0	0.5	1	1.5	SEM	age	dose	age x dose	linear	quadratic
Asp	11.9^a^	10.5^b^	0.17	12.1^a^	11.2^ab^	10.6^b^	10.9^b^	0.24	<0.001	<0.001	0.03	0.01	0.06
Thr	6.5^a^	5.8^b^	0.19	6.7	6.2	5.8	6.0	0.26	0.006	0.120	0.50	0.06	0.23
Ser	7.2^a^	6.5^b^	0.11	7.4^a^	6.9^ab^	6.5^b^	6.7^b^	0.16	<0.001	<0.001	0.01	0.002	0.07
Glu	15.1^a^	12.9^b^	0.19	15.2^a^	14.1^b^	13.2^b^	13.6^b^	0.27	<0.001	<0.001	0.003	0.003	0.06
Pro	4.0^b^	4.7^a^	0.07	4.9^a^	4.4^b^	4.0^c^	4.2^bc^	0.10	<0.001	<0.001	0.08	<0.001	0.01
Gly	4.0^a^	3.6^b^	0.06	4.1^a^	3.8^ab^	3.6^b^	3.8^b^	0.08	<0.001	<0.001	0.02	0.004	0.04
Ala	5.9^a^	5.3^b^	0.08	6.1^a^	5.6^b^	5.3^b^	5.4^b^	0.12	<0.001	<0.001	0.02	<0.001	0.02
Cys	4.9	5.0	0.08	5.1^ab^	5.2^a^	4.7^b^	4.7^b^	0.11	0.43	0.005	0.10	0.003	0.96
Val	6.6^a^	5.9^b^	0.09	6.6^a^	6.3^ab^	6.0^b^	6.2^ab^	0.14	<0.001	0.02	0.04	0.04	0.18
Met	5.4	5.2	0.08	5.6^a^	5.5^a^	5.1^b^	5.0^b^	0.11	0.16	<0.001	0.19	<0.001	0.79
Ile	4.8^a^	4.4^b^	0.07	4.8^a^	4.6^ab^	4.3^b^	4.5^ab^	0.10	<0.001	0.007	0.09	0.01	0.08
Leu	9.6^a^	8. 9^b^	0.14	9.8^a^	9.4^ab^	8.8^b^	9.1^ab^	0.20	<0.001	0.01	0.06	0.01	0.16
Tyr	4.6^a^	4.1^b^	0.07	4.6^a^	4.4^ab^	4.2^b^	4.3^ab^	0.09	<0.001	0.04	0.08	0.06	0.19
Phe	7.0^a^	6.0^b^	0.11	6.9^a^	6.6^ab^	6.2^b^	6.4^ab^	0.15	<0.001	0.01	0.04	0.02	0.18
His	2.8^a^	2.5^b^	0.04	2.8^a^	2.7^ab^	2.5^b^	2.6^ab^	0.06	<0.001	0.01	0.08	0.02	0.10
Lys	7.7^a^	6.9^b^	0.11	7.7^a^	7.3^ab^	7.0^b^	7.2^ab^	0.16	<0.001	0.02	0.02	0.05	0.14
Arg	5.3^a^	4.7^b^	0.08	5.3^a^	5.1^ab^	4.7^b^	4.9^ab^	0.11	<0.001	0.01	0.13	0.02	0.16
Trp	1.3	1.4	0.05	1.3^ab^	1.4^a^	1.5^a^	1.1^b^	0.072	0.090	0.006	<0.001	0.12	0.02

1 Data are presented as least squares means and standard error of the mean (SEM) ^a, b, c^ Means in the same row and for the same main effect (age or dose) with different superscripts are significantly different (*P* < 0.05, Tukey’s HSD test).

2 Linear or quadratic effects were evaluated by orthogonal polynomial contrasts to test the effects of Gln levels on selected traits.

**Table 6:** Amino acid composition of egg yolk (mg/g) as affected by main factors.

TRAIT^1^	Age			Dose					Effect of			Contrast^2^	
	12	19	SEM	0	0.5	1	1.5	SEM	age	dose	age x dose	linear	quadratic
Asp	15.2^b^	15.5^a^	0.10	15.1^b^	16.0^a^	15.2^b^	15.3^b^	0.14	0.04	<0.001	<0.001	0.91	0.02
Thr	7.8^b^	8.4^a^	0.05	7.9^b^	8.5^a^	7.9^b^	8.0^b^	0.08	<0.001	<0.001	<0.001	0.69	0.04
Ser	12.0	12.2	0.09	11.9^b^	12.4^a^	12.1^ab^	12.0^ab^	0.13	0.19	0.03	<0.001	0.83	0.07
Glu	16.7^b^	18.3^a^	0.17	17.4	17.8	17.3	17.4	0.24	<0.001	0.39	<0.001	0.69	0.66
Pro	4.9^b^	5.3^a^	0.04	5.1^b^	5.36^a^	5.1^ab^	4.9^ab^	0.06	<0.001	<0.001	0.041	0.03	0.01
Gly	4.4^b^	4.6^a^	0.03	4.4^b^	4.7^a^	4.5^ab^	4.5^ab^	0.04	<0.001	0.002	<0.001	0.57	0.02
Ala	7.2	7.1	0.05	7.0^b^	7.4^a^	7.1^b^	7.1^ab^	0.07	0.73	0.003	<0.001	0.83	0.04
Cys	3.9^b^	4.1^a^	0.03	3.8^b^	4.1^a^	4.0^ab^	3.9^ab^	0.04	<0.001	0.002	0.002	0.32	0.03
Val	8.1	8.2	0.06	8.0^b^	8.5^a^	8.1^b^	8.1^b^	0.08	0.19	<0.001	0.002	0.76	0.01
Met	8.1^b^	8.8^a^	0.07	8.3	8.5	8.6	8.3	0.09	<0.001	0.08	0.02	0.88	0.07
Ile	7.0^b^	7.4^a^	0.05	7.1^b^	7.5^a^	7.1^b^	7.1^b^	0.07	<0.001	<0.001	0.011	0.20	0.02
Leu	13.1	13.1	0.09	12.8^b^	13.5^a^	13.0^ab^	13.0^ab^	0.13	0.99	0.004	<0.001	0.75	0.02
Tyr	6.6^b^	6.8^a^	0.05	6.6^b^	6.9^a^	6.7^ab^	6.6^b^	0.07	0.002	0.01	0.002	0.87	0.03
Phe	6.4	6.3	0.04	6.2^b^	6.5^a^	6.4^ab^	6.4^ab^	0.06	0.19	0.01	<0.001	0.31	0.04
His	4.2	4.2	0.03	4.0^b^	4.3^a^	4.1^b^	4.1^b^	0.04	0.85	<0.001	0.01	0.88	<0.001
Lys	10.9^b^	12.4^a^	0.31	10.9^b^	13.7^a^	11.0^b^	11.1^b^	0.44	0.001	<0.001	<0.001	0.37	0.02
Arg	9.7^b^	10.2^a^	0.070	9.9^b^	10.3^a^	9.9^b^	9.8^b^	0.09	<0.001	0.01	0.001	0.424	0.106
Trp	2.1	2.2	0.07	2.4^a^	2.2^ab^	1.9^b^	2.1^ab^	0.09	0.76	0.01	0.01	0.040	0.024

1 Data are presented as least squares means and standard error of the mean (SEM) ^a, b^, Means in the same row and for the same main effect (age or dose) with different superscripts are significantly different (*P* < 0.05, Tukey’s HSD test).

2 Linear or quadratic effects were evaluated by orthogonal polynomial contrasts to test the effects of Gln levels on selected traits.

**Table 7:** Amino acid composition of egg albumen (mg/g) for traits showing a significant age × dose interaction.

Age	Dose	TRAIT^1^								
		Asp	Ser	Glu	Gly	Ala	Val	Phe	Lys	Trp
12	0	13.1^a^	8.1^a^	16.9^a^	4.4^a^	6.6^a^	7.0^a^	7.5^a^	8.1^a^	1.4^abc^
	0.5	12.3^ab^	7.4^ab^	15.6^ab^	4.2^ab^	6.1^ab^	7.0^a^	7.4^a^	8.1^a^	1.6^ab^
	1	11.1^bc^	6.7^bc^	13.9^bc^	3.7^bc^	5.4^bc^	6.2^ab^	6.6^ab^	7.3^ab^	1.2^bc^
	1.5	11.2^bc^	6.6^bc^	14.0^bc^	3.8^bc^	5.5^bc^	6.3^ab^	6.5^ab^	7.4^ab^	0.9^c^
19	0	11.0^bc^	6.8^bc^	13.5^c^	3.8^bc^	5.6^bc^	6.2^ab^	6.4^b^	7.3^ab^	1.3^abc^
	0.5	10.1^c^	6.3^c^	12.5^c^	3.5^c^	5.1^c^	5.7^b^	5.8^b^	6.4^b^	1.2^bc^
	1	10.2^c^	6.4^c^	12.5^c^	3.6^c^	5.1^c^	5.7^b^	5.8^b^	6.7^b^	1.7^a^
	1.5	10.7^c^	6.7^bc^	13.2^c^	3.7^bc^	5.4^bc^	6.1^b^	6.2^b^	7.0^b^	1.3^abc^
SEM		0.334	0.221	0.384	0.112	0.169	0.193	0.209	0.227	0.101

1 Data are presented as least squares means and standard error of the mean (SEM) ^a, b, c, d^ Means in the same row and for the same main effect (age or dose) with different superscripts are significantly different (*P* < 0.05, Tukey’s HSD test).

## Results

### *Albumen and Yolk proximate composition*

Bird age significantly affected the proximate composition of egg albumen (Table 2). Albumen moisture was higher at 19 wk of age than at 12 wk of age (*P* = 0.001). In contrast, albumen protein content decreased with age and was lower at 19 wk compared with 12 wk (*P* < 0.001). Ash content of albumen was also lower at 19 wk of age (*P* < 0.001).

Albumen fat content was not affected by age (*P* = 0.23). Available carbohydrate content also did not differ between ages when considering the main effect of age (*P* = 0.78).

The calculated energy value of albumen decreased with age. Energy expressed as kJ/100 g (*P* < 0.001) was lower at 19 wk than at 12 wk of age.

Dietary Gln supplementation affected albumen protein content (*P* = 0.018). The highest protein concentration was observed in the control group, whereas the lowest value was recorded at the 1.5% Gln group. A significant linear response of albumen protein content to increasing dietary Gln levels (*P* = 0.008), with no quadratic effect was revealed.

No significant effects of dietary Gln dose were observed for albumen moisture, fat, ash, or energy value (*P* > 0.05). Available carbohydrate content was not affected by Gln dose as a main effect (*P* = 0.107); however, a significant age × dose interaction was detected for this trait (*P* = 0.005).

Bird age significantly affected the proximate composition of egg yolk (Table 3). Yolk moisture was lower at 19 wk of age than at 12 wk (*P* < 0.001). In contrast, yolk protein content increased with age and was higher at 19 wk compared with 12 wk (*P* = 0.001). Yolk fat content was also higher at 19 wk of age than at 12 wk (*P* < 0.001). Ash content increased with age and was higher in yolks collected at 19 wk (*P* < 0.001).

Available carbohydrate content of yolk was higher at 19 wk than at 12 wk of age (*P* < 0.001). As a result of these compositional changes, the calculated energy value of yolk increased with age. Energy expressed as kJ/100 g (*P* < 0.001) was higher at 19 wk than at 12 wk of age.

Dietary Gln supplementation affected several yolk components. A significant main effect of Gln dose was observed for yolk moisture (*P* = 0.002), with the lowest value recorded at the highest supplementation level (1.5% Gln). Yolk protein content was also affected by Gln dose (*P* < 0.001), with the highest value observed at the 0.5% Gln level. For this trait, a significant quadratic response to increasing dietary Gln levels was detected (*P* < 0.001).

Yolk fat content was influenced by dietary Gln dose (*P* = 0.012). The lowest fat content was observed at the 1.0% Gln level, whereas higher values were recorded in the control group and at 1.5% Gln group. A significant linear response of yolk fat content to increasing Gln levels was observed (*P* = 0.02).

Ash content of yolk was significantly affected by dietary Gln dose (*P* = 0.001). The highest ash content was observed at the 0.5% Gln level, whereas the lowest value occurred at 1.0% Gln. Available carbohydrate content was not affected by Gln dose as a main effect (*P* = 0.54).

Significant age × dose interactions were detected for yolk moisture (*P* = 0.014), fat (*P* = 0.039), ash (*P* < 0.001), available carbohydrates (*P* = 0.039), and energy value expressed as kJ/100 g (*P* = 0.001), indicating that the response of yolk composition to dietary Gln differed between ages.

### *Age × dietary glutamine interactions in yolk composition*

Significant age × dietary Gln interactions were observed for several yolk traits, including moisture, fat content, ash content, available carbohydrates, and energy value expressed as kJ/100 g (*P* < 0.05) (Table 4).

For yolk moisture, a significant interaction indicated a stronger response to dietary Gln at 19 wk of age than at 12 wk. At 12 wk, yolk moisture showed relatively small differences among dietary treatments, whereas at 19 wk a marked decrease in moisture content was observed at the 1.5% Gln level compared with the remaining treatments.

Yolk fat content exhibited an age-dependent response to dietary Gln. At 12 wk of age, the lowest fat content was recorded at the 1.0% Gln level, whereas higher values were observed in the control group and at 1.5% Gln group. At 19 wk of age, yolk fat content remained high across all dietary treatments, with no clear dose-dependent pattern.

Ash content of yolk showed a significant age × dose interaction. At 12 wk of age, ash content decreased progressively with increasing dietary Gln level, reaching the lowest value at 1.5% Gln. In contrast, at 19 wk of age, ash content was highest at the 0.5% and 1.5% Gln levels and lowest at 1.0% Gln.

Available carbohydrate content of yolk was also affected by the interaction between age and dietary Gln. At 12 wk, available carbohydrates increased with increasing Gln level up to 1.0%, followed by a decrease at 1.5%. At 19 wk, the highest carbohydrate content was observed in the control group, whereas supplementation with Gln resulted in lower values across all supplemented treatments.

The calculated energy value of yolk reflected the interaction patterns observed for fat and carbohydrate contents. At 12 wk of age, yolk energy increased with dietary Gln level and reached the highest value at 1.5% Gln. In contrast, at 19 wk of age, yolk energy was highest in the control group and decreased in all Gln-supplemented groups, with the lowest values observed at 1.0% and 1.5% Gln.

### *Amino acid composition of egg albumen - main effects*

The amino acid composition of egg albumen was significantly influenced by bird age (Table 5). For the majority of amino acids, albumen collected at 19 wk of age exhibited lower concentrations compared with albumen collected at 12 wk of age.

Significant age-related decreases were observed for Asp, Thr, Ser, Glu, Gly, Ala, Val, Ile, Leu, Tyr, Phe, His, Lys, and Arg (*P* < 0.05). In contrast, Pro content increased with age and was higher at 19 wk compared with 12 wk (*P* < 0.001). No significant effect of age was observed for Cys, Met, or tryptophan (*P* > 0.05).

Dietary Gln supplementation affected the concentration of several amino acids in egg albumen. A significant main effect of Gln dose was detected for Asp, Ser, Glu, Pro, Gly, Ala, Val, Met, Ile, Leu, Tyr, Phe, His, Lys, Arg, and Trp (*P* < 0.05). For most of these amino acids, the highest concentrations were observed in the control group or at the lowest supplementation level, whereas reduced values were recorded at higher Gln doses.

Significant linear responses to increasing dietary Gln levels for Asp, Ser, Glu, Gly, Ala, Val, Ile, Leu, Phe, His, Lys, Arg, and Trp was observed (*P* < 0.05), indicating a progressive decrease in their concentrations with increasing Gln supplementation. Quadratic responses were detected for Pro, Gly, Ala, and Trp (*P* < 0.05), reflecting non-linear dose-dependent patterns.

No significant main effects of dietary Gln dose were observed for Thr or Cys (*P* > 0.05).

Several amino acids exhibited significant age × dietary Gln interactions, including Asp, Ser, Glu, Gly, Ala, Val, Phe, Lys, and Trp (*P* < 0.05), indicating that the response of albumen amino acid composition to Gln supplementation differed between age groups. These interactions are detailed in Table 7.

### *Amino acid composition of egg yolk - main effects*

Bird age significantly affected the amino acid composition of egg yolk (Table 6). For most amino acids, higher concentrations were observed at 19 wk of age compared with 12 wk.

Significant age-related increases were found for Asp, Thr, Glu, Pro, Gly, Val, Met, Ile, Tyr, Lys, Arg, and Cys (*P* < 0.05). No significant effect of age was detected for Ser, Ala, Leu, Phe, His, or Trp (*P* > 0.05).

Dietary Gln supplementation significantly influenced the yolk amino acid profile. A significant main effect of Gln dose was observed for Asp, Thr, Ser, Pro, Gly, Ala, Val, Ile, Leu, Tyr, Phe, His, Lys, Arg, Trp, and Cys (*P* < 0.05). For most of these amino acids, the highest concentrations were recorded at the 0.5% Gln level, whereas lower values were generally observed in the control group and at higher supplementation levels.

Significant linear responses to increasing dietary Gln levels for Asp, Thr, Ser, Gly, Ala, Val, Ile, Leu, Tyr, Phe, His, Lys, Arg, Trp, and Cys was observed (*P* < 0.05). Quadratic responses were detected for Pro and Gly (*P* < 0.05), reflecting non-linear dose-dependent changes in yolk amino acid concentrations (Table 8).

**Table 8:** Amino acid composition of egg yolk (mg/g) for traits showing a significant age × dose interaction.

Age	Dose	TRAIT^1^																	
		Asp	Thr	Ser	Glu	Pro	Gly	Ala	Cys	Val	Met	Ile	Leu	Tyr	Phe	His	Lys	Arg	Trp
12	0	14.8^b^	7.6^c^	11.8^b^	16.9^bc^	5.0^bc^	4.3^b^	7.0^bc^	3.8^b^	7.9^b^	7.8^e^	6.9^b^	12.7^b^	6.5^b^	6.2^bc^	4.0^c^	10.8^b^	9.6^b^	2.5^a^
	0.5	15.3^b^	7.8^bc^	11.8^b^	15.9^c^	5.1^bc^	4.4^b^	7.1^bc^	3.9^ab^	8.2^b^	8.0^de^	7.2^b^	12.9^b^	6.5^b^	6.3^bc^	4.3^ab^	10.8^b^	9.7^b^	2.3^a^
	1	15.2^b^	7.7^c^	12.2^b^	16.8^bc^	5.1^bc^	4.5^b^	7.2^bc^	3.7^b^	8.1^b^	8.4^bcde^	7.0^b^	13.1^ab^	6.6^b^	6.5^abc^	4.2^bc^	10.9^b^	9.7^b^	1.6^b^
	1.5	15.7^b^	8.0^bc^	12.3^ab^	16.9^bc^	4.8^c^	4.5^b^	7.3^ab^	4.0^ab^	8.3^b^	8.2^cde^	7.1^b^	13.4^ab^	6.7^b^	6.6^ab^	4.2^abc^	11.2^b^	9.8^b^	2.1^ab^
19	0	15.3^b^	8.2^b^	12.0^b^	17.9^b^	5.2^b^	4.5^b^	7.0^bc^	3.9^b^	8.1^b^	8. 8^abc^	7.3^b^	12.8^b^	6.7^b^	6.2^c^	4.1^bc^	11.1^b^	10.1^b^	2.2^a^
	0.5	16.7^a^	9.1^a^	13.0^a^	19.7^a^	5.6^a^	4.9^a^	7.6^a^	4.2^a^	8.8^a^	9.1^a^	7. 9^a^	13.9^a^	7.2^a^	6.8^a^	4.4^a^	16.6^a^	10.9^a^	2.0^ab^
	1	15.3^b^	8.2^b^	11.9^b^	17.6^b^	5.2^b^	4.6^b^	7.0^bc^	4.2^a^	8.1^b^	8.8^ab^	7.2^b^	12.9^b^	6.7^b^	6.2^bc^	4.1^bc^	11.1^b^	10.0^b^	2.2^ab^
	1.5	14.9^b^	8.0^bc^	11.7^b^	17.8^b^	5.1^b^	4.5^b^	6.9^c^	4.0^ab^	7.9^b^	8.5^bcd^	7.1^b^	12.6^b^	6.6^b^	6.1^c^	4.0^c^	10.9^b^	9.9^b^	2.2^a^
SEM		0.206	0.109	0.179	0.347	0.079	0.063	0.098	0.062	0.112	0.139	0.102	0.186	0.098	0.089	0.054	0.621	0.141	0.135

1 Data are presented as least squares means and standard error of the mean (SEM) ^a, b, c, d^ Means in the same row and for the same main effect (age or dose) with different superscripts are significantly different (*P* < 0.05, Tukey’s HSD test).

No significant main effect of dietary Gln dose was observed for Glu or methionine (*P* > 0.05).

Several amino acids exhibited significant age × dietary Gln interactions, including Asp, Thr, Ser, Glu, Pro, Gly, Ala, Val, Met, Ile, Leu, Tyr, Phe, His, Lys, Arg, and Trp (*P* < 0.05), indicating that the response of yolk amino acid composition to dietary Gln differed between age groups. These interactions are presented in detail in Table 7.

### *Age × dietary glutamine interactions for amino acid composition of egg albumen*

Significant age × dietary Gln interactions were detected for several amino acids in egg albumen, including Asp, Ser, Glu, Gly, Ala, Val, Phe, Lys, and Trp (*P* < 0.05).

For Asp, Ser, and Glu, a clear age-dependent response to dietary Gln was observed. At 12 wk of age, the concentrations of these amino acids decreased progressively with increasing dietary Gln level, with the lowest values generally recorded at the 1.0% and 1.5% supplementation levels. At 19 wk of age, albumen concentrations of Asp, Ser, and Glu were lower overall and showed less pronounced differences among dietary treatments.

Gly and Ala exhibited similar interaction patterns. At 12 wk, the highest concentrations were observed in the control group, followed by a gradual decrease with increasing Gln dose. In contrast, at 19 wk of age, Gly and Ala concentrations remained relatively stable across dietary treatments, with only minor differences among Gln levels.

For Val and Phe, dietary Gln supplementation affected albumen concentrations predominantly at 12 wk of age. At this age, higher concentrations were observed in the control group and at the lowest supplementation level, whereas reduced values were recorded at 1.0% and 1.5% Gln. At 19 wk of age, no clear dose-related pattern was evident.

Lys content of albumen showed a significant age × dose interaction characterized by a stronger response to dietary Gln at 12 wk of age. At this time point, Lys concentration decreased with increasing Gln level, whereas at 19 wk of age Lys content remained relatively uniform across dietary treatments.

For Trp, an interaction between age and dietary Gln was evident. At 12 wk of age, Trp concentration was highest in the control group and decreased markedly with increasing Gln dose. At 19 wk of age, Trp concentrations were generally higher and exhibited a less consistent response to dietary Gln supplementation.

### *Age × dietary glutamine interactions for amino acid composition of egg yolk*

Significant age × dietary Gln interactions were detected for all amino acids analyzed in egg yolk (*P* < 0.05), indicating that the response of yolk amino acid composition to dietary Gln supplementation differed markedly between age groups.

At 12 wk of age, dietary Gln supplementation resulted in relatively moderate changes in yolk amino acid concentrations. For most amino acids, including Asp, Thr, Ser, Glu, Pro, Gly, Ala, Val, Met, Ile, Leu, Tyr, Phe, His, Lys, Arg, and Trp, the highest concentrations were generally observed at intermediate Gln levels (0.5% or 1.0%), whereas lower values were recorded in the control group or at the highest supplementation level (1.5% Gln). The response patterns were non-linear, with no consistent monotonic increase or decrease across Gln doses.

In contrast, at 19 wk of age, yolk amino acid composition exhibited a more pronounced and uniform response to dietary Gln. For the majority of amino acids, including Asp, Thr, Ser, Glu, Pro, Gly, Ala, Val, Met, Ile, Leu, Tyr, Phe, His, Lys, and Arg, the highest concentrations were observed at the 0.5% Gln level. Supplementation with higher Gln doses (1.0% and 1.5%) resulted in reduced concentrations of these amino acids, approaching or falling below values observed in the control group.

Tryptophan exhibited a distinct interaction pattern. At 12 wk of age, Trp concentration was highest in the control group and decreased with increasing dietary Gln level, reaching the lowest value at 1.0% supplementation. At 19 wk of age, Trp concentration peaked at the 0.5% Gln level and declined at higher supplementation levels.

Overall, the age × dietary Gln interactions observed for yolk amino acids indicate age-dependent differences in the magnitude and direction of the response to Gln supplementation, with more pronounced and consistent effects observed in late peak-lay birds.

## Discussion

The present study demonstrates that both dietary Gln supplementation and bird age significantly influenced egg composition in Japanese quails. Clear main effects of age and Gln dose, as well as significant age × Gln interactions, were observed for proximate composition and amino acid profiles of both albumen and yolk. These findings are consistent with previous studies showing that egg composition changes dynamically during the laying cycle and are closely associated with the physiological maturation of the reproductive system. Older quails deposited greater amounts of several nutrients, including protein, fat, and ash, into the egg yolk, which agrees with previous observations in Japanese quails (Nowaczewski et al., 2021). Similarly, age-related changes in egg composition have also been described in both quails and laying hens (Zita et al., 2013; Chang et al., 2024). In the present study, eggs collected at 19 wk of age contained higher yolk protein and fat concentrations than eggs collected at 12 wk, indicating progressive changes in nutrient partitioning during the laying period. These physiological changes likely reflect increasing metabolic and reproductive maturity of the hens and confirm that even within the peak laying phase, bird age remains an important determinant of egg composition.

Against this physiological background, dietary Gln supplementation acted as an additional factor modulating egg composition. The response to Gln was compartment-specific and depended on both bird age and dietary inclusion level. Albumen amino acid composition appeared to be more responsive to dietary Gln than yolk composition, although the direction and magnitude of these changes were not uniform across amino acids or supplementation levels. In albumen, several amino acids decreased progressively with increasing dietary Gln levels, particularly at 1.0% and 1.5% supplementation, whereas moderate supplementation levels occasionally resulted in higher concentrations of selected amino acids. In contrast, yolk composition showed less pronounced responses at the level of proximate composition, although age-dependent changes in yolk amino acid deposition were evident. Similar observations were reported previously in laying hens supplemented with dietary Gln, where modifications in albumen composition occurred without major changes in basic yolk traits or egg component proportions (Tomaszewska et al., 2021). Collectively, these findings suggest that albumen protein deposition may be more sensitive to changes in dietary amino acid availability than yolk formation, which depends primarily on hepatic synthesis and transport of yolk precursors (Nys et al., 2011). Importantly, no changes in egg weight or yolk-to-albumen ratio were observed, indicating that dietary Gln primarily affected nutrient partitioning and amino acid deposition rather than overall egg size or egg component yield.

The reduction in albumen crude protein concentration observed with increasing dietary Gln should therefore be interpreted as a relative compositional change rather than as evidence of impaired albumen formation. Albumen protein concentration depends not only on total nitrogen supply, but also on the balance of amino acids available for oviductal protein synthesis and on the allocation of amino acids between maintenance metabolism and egg formation. Because Gln is an important amino-group donor and participates in amino acid interconversion, additional dietary Gln may have modified nitrogen flux and the utilization of other amino acids without proportionally increasing albumen protein deposition. At higher inclusion levels, this could have resulted in a relative imbalance among amino acids required for albumen protein synthesis, particularly if essential amino acids became limiting in relation to the increased availability of Gln. This interpretation is consistent with the linear decrease in albumen crude protein concentration, while the lack of changes in egg weight and yolk-to-albumen ratio suggests that the effect reflected altered nutrient partitioning rather than impaired egg formation.

The observed changes in albumen amino acid composition following dietary Gln supplementation further suggest that Gln may influence amino acid metabolism and nutrient partitioning during egg formation. However, the response patterns were not uniform and depended on both the amino acid analyzed and the level of dietary supplementation. Previous studies demonstrated that dietary and genetic factors can modify amino acid deposition in egg components, including both yolk and albumen, indicating that egg amino acid composition remains responsive to nutritional and physiological modulation (Mori et al., 2020). One possible explanation involves the central metabolic role of Gln in nitrogen transfer and amino acid interconversion. Glutamine and glutamate are closely linked to α-ketoglutarate metabolism within the tricarboxylic acid cycle and participate in multiple transamination pathways involved in the synthesis of non-essential amino acids, including proline and aspartate-derived compounds. Therefore, dietary Gln supplementation may alter intracellular amino acid fluxes and indirectly influence the deposition of amino acids into albumen proteins. At the same time, the predominantly non-linear responses observed in the present study indicate that excessive dietary Gln does not necessarily promote proportional increases in albumen amino acid concentrations. This suggests that albumen amino acid deposition may be regulated not only by amino acid availability itself, but also by metabolic utilization, amino acid balance, and physiological limitations associated with protein synthesis in the oviduct. Glutamine may also influence albumen composition indirectly through its effects on intestinal metabolism and nitrogen utilization. Because Gln serves as an important carrier of amino groups and an energy substrate for enterocytes and immune cells, dietary supplementation could modify the allocation of other amino acids between maintenance functions and productive processes such as egg formation. Nevertheless, the biological interpretation of these findings should remain cautious. Although statistically significant shifts in albumen amino acid composition were observed, these changes reflected relatively moderate alterations in amino acid proportions rather than major improvements in egg nutritional value. Importantly, no evidence was found that dietary Gln overcame limitations related to essential amino acid balance in albumen protein composition.

The relatively stable Lys concentration in albumen at 19 wk of age may be explained by the essential nature of Lys and by the tighter regulation of albumen protein synthesis during the established laying phase. Unlike non-essential amino acids involved in Gln-dependent transamination pathways, Lys cannot be synthesized from Gln or glutamate. Moreover, all experimental diets were formulated to provide the same Lys content. Therefore, dietary Gln supplementation would not be expected to directly increase Lys availability, but could influence Lys deposition only indirectly through changes in amino acid utilization or oviductal protein synthesis. The lack of a clear dose-related Lys response at 19 wk suggests that Lys incorporation into albumen proteins was relatively conserved in physiologically mature laying quails. In contrast, the stronger decrease in albumen Lys at 12 wk may reflect greater metabolic plasticity during the early laying phase, when oviductal function and nutrient partitioning are still stabilizing.

Compared with albumen, yolk proximate composition was less responsive to dietary Gln supplementation, which is consistent with the physiological mechanisms of yolk formation. Yolk deposition depends largely on hepatic synthesis of vitellogenin and very-low-density lipoproteins and therefore may be less directly affected by short-term changes in dietary amino acid supply (Nys et al., 2011). However, despite the relatively limited effects on yolk macronutrient composition, clear age-dependent changes in yolk amino acid deposition were observed. In particular, moderate dietary Gln inclusion levels were associated with higher concentrations of several amino acids, whereas higher supplementation levels frequently resulted in reduced values. These findings indicate that yolk amino acid composition remains metabolically responsive to dietary manipulation, although the response appears strongly dependent on both bird age and Gln dose. The present observations are consistent with previous studies showing that dietary interventions may alter amino acid deposition into egg yolk. Supplementation with HMB modified yolk amino acid composition in laying hens (Dajnowska et al., 2023), whereas nutritional strategies improving protein digestibility were associated with altered yolk amino acid concentrations (Horniaková and Michálková, 2007). Similarly, walnut meal supplementation increased selected amino acids in yolk (Bai et al., 2025). Collectively, these findings support the concept that dietary modulation of amino acid metabolism can influence yolk amino acid composition, although the response is selective and highly dependent on metabolic and physiological context.

The significant age × Gln interactions observed in the present study indicate that the response to dietary Gln was strongly dependent on the physiological stage of the birds. Quails at 12 and 19 wk of age responded differently to the same dietary Gln levels, suggesting age-dependent differences in nutrient utilization and metabolic priorities. During the early laying phase, younger birds are still completing physiological maturation while simultaneously supporting intensive egg production. In contrast, late peak-lay birds at 19 wk of age have fully established reproductive activity and may exhibit different patterns of nutrient partitioning associated with prolonged laying activity and metabolic adaptation.

Because Gln becomes conditionally essential under periods of elevated metabolic demand or physiological stress, its effects may depend on the baseline metabolic state of the birds. It is possible that younger quails already maintained relatively efficient amino acid utilization, thereby limiting the response to additional dietary Gln. Conversely, late peak-lay birds may exhibit different patterns of nutrient partitioning associated with prolonged reproductive activity and metabolic adaptation. Similar age-related alterations in albumen synthesis and oviduct function have been described previously in laying hens (Chang et al., 2024). Although the present study did not directly evaluate the mechanisms involved, the stronger responses observed in late peak-lay birds support the concept that physiological age may influence sensitivity to dietary amino acid supplementation.

Another possible explanation for the age-dependent response involves differences in nutrient utilization efficiency. Glutamine is a major metabolic substrate for enterocytes and immune cells and plays an important role in maintaining gut integrity and immune function (Bartell and Batal, 2007). If late peak-lay quails experienced greater metabolic demands associated with sustained egg production, dietary Gln supplementation may have contributed to maintaining nutrient utilization efficiency and amino acid availability for egg formation. However, because intestinal and immune parameters were not evaluated in the present study, this interpretation should be considered speculative and requires further investigation.

Research investigating dietary Gln supplementation in laying birds remains relatively limited. Previous studies in heat-stressed laying hens demonstrated that dietary Gln supplementation helped maintain productive performance under thermal stress conditions (Zavarize et al., 2011), suggesting that Gln may support physiological resilience during periods of increased metabolic challenge. Similarly, studies conducted in guinea fowl breeders showed that dietary Gln supplementation improved selected egg quality traits, including shell strength and hatchability, without affecting egg weight or the proportions of internal egg components (Gholipour et al., 2017). These observations are consistent with the present results, in which dietary Gln exerted more evident effects on compositional and qualitative traits than on gross egg characteristics.

Likewise, Muszyński et al. (2023) demonstrated that dietary Gln supplementation increased eggshell thickness and calcium deposition without affecting egg size. Together with the present findings, these observations suggest that Gln may primarily influence nutrient partitioning and selected egg quality traits rather than overall egg mass deposition. Effects of dietary Gln appear to be more evident at the level of compositional characteristics than gross egg production traits.

The present results therefore support the view that Gln acts primarily as a functional metabolic modulator rather than a direct stimulator of egg mass production. Its effects appear to involve subtle alterations in nutrient utilization and amino acid deposition that depend on physiological state, bird age, and dietary inclusion level.

However, although many of the observed changes in egg composition were statistically significant, their magnitude remained relatively moderate. The present findings therefore indicate selective modulation of egg composition rather than a substantial improvement in overall egg nutritional value. From a physiological perspective, dietary Gln supplementation appears to influence nutrient partitioning and amino acid deposition during egg formation, particularly in relation to bird age and laying stage. Importantly, no adverse effects of dietary Gln supplementation on egg composition or bird performance were observed within the supplementation range used in the present study.

The age-dependent responses observed in this study may have implications for dietary formulation strategies during different phases of the laying cycle. The results suggest that the response to dietary Gln depends on physiological stage and metabolic status, indicating that the effectiveness of supplementation may vary between early and later phases of egg production. Such observations are consistent with the concept of phase-specific nutritional management in laying birds. In addition, because Gln is closely associated with nitrogen metabolism and amino acid interconversion, its effects may depend on the overall amino acid balance of the diet. Previous studies emphasized the importance of maintaining appropriate essential amino acid supply in laying hens to support efficient protein deposition and egg formation (Selle et al., 2024; Macelline et al., 2021). Therefore, the response to dietary Gln supplementation should likely be interpreted within the broader context of dietary protein and amino acid composition.

Future studies should further investigate the biological mechanisms underlying the observed responses to dietary Gln supplementation. In particular, studies evaluating oviduct metabolism, protein synthesis pathways, intestinal physiology, and circulating amino acid profiles could help clarify how Gln influences egg component formation during different stages of lay. Additional dose-response studies would also be valuable to determine the optimal supplementation range for laying birds. Previous studies in broilers demonstrated that excessive dietary Gln levels may negatively affect performance (Bartell and Batal, 2007), indicating that both efficacy and safety should be considered when evaluating practical applications in laying poultry.

In conclusion, dietary l-glutamine did not uniformly enhance the nutritional composition of quail eggs, but selectively modulated amino acid deposition in an age- and dose-dependent manner. The response was more evident in amino acid profiles than in conventional proximate composition, indicating that Gln acted primarily as a regulator of nutrient partitioning rather than as a direct enhancer of gross egg nutrient content. Albumen showed changes consistent with altered amino acid allocation during albumen formation, whereas yolk responses were more dependent on bird age, probably reflecting hepatic and reproductive regulation of yolk precursor deposition. The most consistent responses were generally observed at moderate Gln inclusion levels, particularly 0.5% and 1.0%, whereas 1.5% Gln did not provide consistent additional benefits. These findings suggest that Gln supplementation in laying quails may be considered as a phase-specific and dose-dependent nutritional strategy, with potential relevance for modulating egg amino acid composition during different stages of lay.

## Author credit statement

Ewa Tomaszewska: Conceptualization, Data Curation, Writing – Original Draft, Writing – Review and Editing, Supervision, Funding acquisition.

Kornel Kasperek: Conceptualization, Formal analysis, Writing – Review and Editing, Supervision.

Kamil Drabik: Conceptualization, Data Curation, Writing – Review and Editing.

Iwona Puzio: Investigation, Resources, Data Curation, Writing – Original Draft.

Katarzyna J. Zelewska: Data Curation, Writing – Original Draft.

Justyna Batkowska: Investigation, Resources, Writing – Original Draft.

## Disclosures

The authors declare that they have no conflicts of interest.

## References

[bib0001] AOAC International (2019).

[bib0002] Bai X., Bi J., Li A., Deng X., Zhao Z., Hu H., Pan H. (2025). Walnut cake meal improves amino acids, fatty acid composition and flavor of egg yolk via the microbiota-yolk metabolites crosstalk in Jingfen-1 laying hens. Poult. Sci..

[bib0003] Bartell S.M., Batal A.B. (2007). The effect of supplemental glutamine on growth performance, development of the gastrointestinal tract, and humoral immune response of broilers. Poult. Sci..

[bib0004] Chang X.Y., Edna O.U., Wang J., Zhang H.J., Zhou J.M., Qiu K., Wu S.G. (2024). Histological and molecular difference in albumen quality between post-adolescent hens and aged hens. Poult. Sci..

[bib0005] Dajnowska A., Tomaszewska E., Świątkiewicz S., Arczewska-Włosek A., Dobrowolski P., Domaradzki P., Rudyk H., Brezvyn O., Muzyka V., Kotsyumbas I., Arciszewski M.B., Muszyński S. (2023). Yolk fatty acid profile and amino acid composition in eggs from hens supplemented with ß-hydroxy-ß-methylbutyrate. Foods.

[bib0006] Gholipour V., Chamani M., Shahryar H.A., Sadeghi A., Afshar M.A. (2017). Effects of dietary L-glutamine supplement on performance, egg quality, fertility and some blood biochemical parameters in Guinea fowls (Numida meleagris). Kafkas Univ. Vet. Fak. Derg..

[bib0007] Horniaková E., Michálková J. (2007). Effect of diet on amino acid profile of egg yolk. Pol. J. Food Nutr. Sci..

[bib0008] Macelline S.P., Toghyani M., Chrystal P.V., Selle P.H., Liu S.Y. (2021). Amino acid requirements for laying hens: a comprehensive review. Poult. Sci..

[bib0010] Mori H., Takaya M., Nishimura K., Goto T. (2019). Breed and feed affect amino acid contents of egg yolk and eggshell color in chickens. Poult. Sci..

[bib0011] Muszyński S., Tomaszewska E., Arczewska-Włosek A., Kasperek K., Batkowska J., Lamorski K., Wiącek D., Donaldson J., Świątkiewicz S. (2023). Dietary L-glutamine affects eggshell quality in the post-peak laying period. Ann. Anim. Sci..

[bib0012] Nowaczewski S., Szablewski T., Cegielska-Radziejewska R., Stuper-Szablewska K., Rudzińska M., Tomczyk Ł., Michalak M., Wolc A., Hejdysz M. (2021). Effect of age of Japanese quail on physical and biochemical characteristics of eggs. S. Afr. J. Anim. Sci..

[bib0013] Nys Y., Bain M., Van Immerseel F. (2011). Improving the Safety and Quality of Eggs and Egg Products.

[bib0014] Selle P.H., Macelline S.P., Toghyani M., Liu S.Y. (2024). The potential of glutamine supplementation in reduced-crude protein diets for chicken-meat production. Anim. Nutr..

[bib0016] Tomaszewska E., Arczewska-Włosek A., Burmańczuk A., Pyz-Łukasik R., Donaldson J., Muszyński S., Świątkiewicz S. (2021). The effect of L-glutamine on basal albumen and yolk indices, and albumen amino acids composition. Animals.

[bib0017] Tomaszewska E., Drabik K., Kasperek K., Dobrowolski P., Hułas-Stasiak M., Pyz-Łukasik R., Arciszewski M.B., Muszyński S. (2025). Evaluating the effects of dietary glutamine on performance, carcass traits, blood biochemistry, and intestine morphology in laying quail. Poult. Sci..

[bib0018] Walker S., Baum J.I. (2022). Eggs as an affordable source of nutrients for adults and children living in food-insecure environments. Nutr. Rev..

[bib0019] Wu G. (2013). Functional amino acids in nutrition and health. Amin. Acid..

[bib0021] Zavarize K.C., Sartori J.R., Pezzato A.C., Garcia E.A., Cruz V.C. (2011). Glutamine in diet of laying hens submitted to heat stress and thermoneutrality. Rev. Bras. Zootec..

[bib0022] Zita L., Ledvinka Z., Klesalová L. (2013). The effect of the age of Japanese quails on certain egg quality traits and their relationships. Vet. Arh..

